# Red cell cryopreservation for serologic testing: bulk freezing with polyvinylpyrrolidone (PVP)

**DOI:** 10.55730/1300-0144.6193

**Published:** 2025-10-13

**Authors:** Dilek GÜRLEK GÖKÇEBAY, Willy Albert FLEGEL

**Affiliations:** 1 Department of Transfusion Medicine, NIH Clinical Center, National Institutes of Health, Bethesda, MD, USA; 2 Department of Pediatric Hematology Oncology, University of Health Sciences Ankara Bilkent City Hospital, Ankara, Turkiye

**Keywords:** Immunohematology, laboratory techniques, thawing temperature, defrosting, long-term storage

## Abstract

**Background/aim:**

Red cell cryopreservation is essential for immunohematology reference laboratories. Various cryopreservation methods maintain the structural integrity of red cells, such as bulk freezing with glycerol and droplet freezing with polyvinylpyrrolidone (PVP). In this paper, we provide a technical note with real-world data and report our experience with a modified PVP bulk-freezing method in a transfusion medicine setting.

**Materials and methods:**

Red cells were separated from whole blood samples and incubated for 1 h at room temperature with PVP with slow agitation or no agitation before freezing. Instead of using the established method of shock freezing droplets in liquid nitrogen, the red cells were frozen as 1 mL bulk samples. Recovery rates were determined for different thawing temperatures.

**Results:**

The overall red cell recovery rate was 45%±9% (mean±standard deviation). The recovery rates were 42.3%±8.1% in samples incubated without agitation (n = 27) and 47.9%±9.3% with slow agitation (n = 24) before freezing (p = 0.017). The recovery rates at different thawing temperatures, were 34.0%±2.0% at 22 °C, 46.6%±9.3% at 37 °C, and 40.8%±4.9% at 42°C (p=0.007). A generalized linear model confirmed that slow agitation during incubation yielded higher recovery than no agitation (p = 0.029).

**Conclusion:**

Bulk freezing of red cells with PVP in liquid nitrogen yielded 45% recovery. Our modified method may be preferred for cryopreservation and storage of large volumes. Bulk freezing with PVP can be particularly useful in applications where maximizing storage efficiency is more important than minimizing cell loss.

## 1. Introduction

Red cell cryopreservation is essential for many immunohematology reference laboratories. Long-term storage of blood samples with rare antigens is needed for antibody identification. Cryoprotective agents are required [1] to avert injury of the red cells during the freezing and thawing processes [2,3]. A range of nonpermeating and permeating cryoprotective agents, including polyvinylpyrrolidone (PVP) [4–7], various glycols [8], sugars [9], and hydroxyethyl starch [3,8] have been evaluated and applied since the 1960s [10]. Standard cryopreservation methodologies used in reference laboratories today include gradual freezing (1 °C/min) at −80 °C of bulk samples with glycerol [2,3] and rapid freezing in liquid nitrogen of droplets with PVP [11,12]. Both techniques aim to preserve red cell membrane integrity and to ensure high recovery rates after thawing [3,10].

During freezing, the formation of extracellular ice excludes water, increasing the concentration of nonpenetrating solutes (substances in solution). This creates a hyperosmotic environment that induces osmotic water efflux from red cells. The resulting cellular dehydration and transmembrane pressure differentials compromise red cell membrane integrity. Furthermore, mechanical interactions between cells and ice crystals contribute additional structural damage [13–16].

Glycerol penetrates red cells and prevents excessive dehydration and any critical concentration of the intracellular solution during gradual freezing [3]. Bulk freezing with glycerol in low or high concentration are the standard methods to preserve red cell units for transfusion to patients [2].

PVP extracellularly minimizes ice formation, enabling rapid freezing before the intracellular concentration becomes detrimental [3,7,6]. The significance of freezing rate and temperature gradient for maximal survival of red cells during PVP freezing was recognized early on [17]. Additionally, stagnant thawing in a water bath at temperatures between 37°C and 45°C has yielded up to 97% red cell recovery [18]. Therefore, droplet freezing with PVP has become a preferred method to preserve red cells for serologic testing [12].

Red cells retain their antigenic features for serologic testing after being frozen in storage [19,20]. Droplet-frozen red cells maintain their antigenic reactivity to common blood group antigens comparable to that of fresh red cells [21].

The PVP protective mechanism requires rapid freezing, for example by liquid nitrogen [7,11], whereas the glycerol protective mechanism does not [3]. Despite their effectiveness, both methods have specific limitations. Bulk freezing with glycerol is simple, allowing to use the more readily available −80 °C freezers, but the required deglycerolizing after thawing is labor intensive and time consuming [22]. Droplet freezing with PVP is labor intensive and storage of the droplet occupies larger volumes, while the thawing is rapid and simple [7,12].

Bulk freezing with PVP may offer a high-throughput alternative for cryopreservation of larger volumes of red cells. This may be useful in situations when maximizing storage efficiency is more important than minimizing red cell loss. We report on our experience using bulk freezing with PVP, applied to residual patient and donor sample material, and provide real-world data on this modified method.

## 2. Materials and methods

### Blood samples

We conducted a prospective observational study, reported separately, evaluating regularly transfused pediatric patients with β-thalassemia major. EDTA-anticoagulated whole blood samples from patients were collected at Ankara Bilkent City Hospital Pediatric Hematology outpatient clinic and shipped to the NIH Clinical Center. All samples arrived within 4 days with temperatures ranging from 4 °C to 24 °C and then stored at 4 °C until processing 21 to 27 days after blood collection. The red cells were separated by centrifugation at 1000 rpm for 5 min and washed 3 to 5 times in saline until no hemolysis was observed in the supernatant. Most red cells were droplet frozen with PVP using the conventional method [12]. The remaining red cells were bulk frozen with PVP (Table S1). Six fresh red cell samples from healthy blood donors were also separated using EDTA-anticoagulated whole blood samples (Table S1).

### Bulk freezing with PVP

Bulk freezing with PVP is a modification of the droplet freezing method with PVP. A standard operation procedure (SOP) was previously published [12] for the PVP freezing solution consisting of 23 mL of 30% PVP (average molecular weight 40,000 Da; Sigma-Aldrich, St. Louis, MO, USA) in deionized water and 27 mL of 30% bovine serum albumin. Equal volumes of 0.5 mL packed red cells and 0.5 mL PVP freezing solution were mixed for to total volume of 1 mL red cell suspension in 1.8 mL cryovials that were labeled for each patient.

Randomly chosen cryovials were either placed stationary in a rack (no agitation) or in a benchtop shaking incubator (LSE; Corning, Glendale, AZ, USA) set to 20 revolutions per minute (slow agitation) for the duration of the incubation. The hematocrit (HCT) was determined using a microhematocrit centrifuge (EKF Diagnostics, Boerne, TX, USA) after 1 h incubation at room temperature (HCT before freezing). The cryovials were then immersed in liquid nitrogen at −192 °C and kept in liquid nitrogen vapor at approximately −180 °C inside a liquid nitrogen tank for long-term storage.

### Thawing

A cryovial was removed from the liquid nitrogen tank, unscrewed, and submerged in a 50 mL conical tube containing 30 mL of saline at a given temperature. The conical tube was closed with the cap and gently inverted for 1 min to thaw and suspend the red cells. The empty cryovial was removed from the conical tube and discarded. The conical tube was then centrifugated at 1000 rpm for 20 min. The supernatant that appeared red due to hemolysis was aspirated and discarded. After adding 30 mL of saline solution at room temperature, the conical tube was centrifugated at 1000 rpm for 20 min. This washing process was repeated 3 to 5 times until the supernatant was clear. Once the supernatant was clear, it was removed and discarded. The volume of the red cell pellet was measured with a pipette, recorded, and saline was added (HCT after thawing).

We calculated the red cell recovery using the following formula:


$$\text{Recovery }(\%)=\frac{\text{HCT after thawing }(\%)}{\text{HCT before freezing }(\%)}\times 100$$

modified from Schmid et al. [12]. As a working example (sample number 1 in Table S1), we calculated a recovery rate of 43% for a 500 μL sample of red cell pellet (total volume 1 mL at HCT 46.5% before freezing) resulting in a 300 μL sample of red cell pellet (total volume 1 mL at HCT 20% after thawing): 20/46.5 × 100 = 43%.

### Statistics

Analysis of data was primarily descriptive using mean (SD) for continuous variables (Tables 1 and 2). Data was checked for normality using the Kolmogorov–Smirnov test. The three samples thawed at 40, 45, and 50 °C were not included in the statistical analysis. Comparisons between groups were conducted with nonparametric tests (Mann–Whitney U test for 2 groups and Kruskal–Wallis test for more than 2 groups), because the distribution of the data was not normal (Table 1). A generalized linear model (GLM) was used to assess the effects of multiple independent variables (Table 2) on the continuous dependent variable (Table S2); GLMs extend traditional linear models, such as ANOVA and regression, to allow analysis of nonnormal response distributions. All analyses were performed with SPSS version 27 (IBM, Armonk, NY, USA). P less than 0.05 were considered as statistically significant.

### Antigen typing

Genomic DNA was isolated from the buffy coat (Qiagen EZ1 DNA blood kit on the BioRobot EZ, Qiagen, Valencia, CA, USA), and red cell genotyping was performed using a commercial kit (PreciseType HEA, Immucor, Warren, NJ, USA). Representative red cells from 8 patients were serologically tested for the presence of molecularly predicted antigens (Tables S3 and S4) using standard serology techniques including an adsorption/elution method [23]. Commercial monoclonal or polyclonal reagents and human sera with anti-V, anti-U, or anti-Lu^a^ from stored patient samples were used.

## 3. Results and Discussion

Conventional red cell freezing methods for immunohematology laboratories use bulk freezing with glycerol, which necessitates a lengthy deglycerolizing process. Alternatively, droplet freezing with PVP is often used, which necessitates more storage capacity. Notably, bulk freezing with PVP is not widely used and a standardized protocol has not been established. Bulk freezing with PVP may be preferable when managing large sample volumes under limited storage capacity. To address this gap, we aimed to evaluate bulk freezing with PVP as a modification of the method that we had previously published [12].

This brief technical note can hardly add to the many works on the topic, particularly performed by research groups until 1970 [10]. We used residual red cell samples from pediatric patients with β-thalassemia major to explore bulk freezing with PVP and confirmed that bulk freezing with PVP, despite its lower recovery rate, may be adopted as an adjunct method to droplet freezing with PVP. As a consequence of our study, we will adjust our SOP [12] to include a protocol for bulk freezing with PVP—an incremental step to improve clinical laboratory efficacy. A total of 48 patient samples and 6 random donor samples (Table S1, https://doi.org/10.48623/aperta.286319) were included in the study to provide real-world data on bulk freezing of red cells with PVP.

Schmid et al. [12] reported red cell recovery rates of 85% for droplet freezing with PVP, 41% (16) for bulk glycerol freezing, and 56% (11) with droplet freezing in sucrose and dextrose. We found a postthaw recovery rate of approximately 45% (Table 1). This recovery rate would be unacceptable for transfusable red cell units. However, this recovery rate is sufficient for most laboratory testing purposes. The lower recovery rate may be partially explained by our samples from a cohort of patients who have been transfused every 3 to 4 weeks. Some of their circulating red cells are allogeneic from blood donors and have acquired cellular damage during their storage as blood units before being transfused. This should be considered for evaluations involving red cells from chronically transfused patient populations.

Several technical aspects influenced the overall recovery rate of 45%. Our current protocol [12] stipulated the incubation of red cells and PVP solution without agitation. A protocol modification using slow agitation improved the recovery rate significantly (Table 1). This may be caused by more uniform coating of red cells with PVP as a cryoprotectant. Evaluating various temperatures (22 °C and 42 °C) confirmed that our current protocol using 37 °C gives the best results (Table 1). Higher temperatures of 45 °C and 50 °C hinder recovery (Table S1, https://doi.org/10.48623/aperta.286319). We analyzed the combined effects of 2 incubation conditions and 3 thawing temperatures on red cell recovery (Table 2). The GLM showed that slow agitation during incubation resulted in significantly higher red cell recovery compared with no agitation (B = −4.88, 95% CI −9.28 to −0.49, p = 0.029) (Table S2, https://doi.org/10.48623/aperta.286319), while thawing temperature had no significant effect on recovery.

Red cells preserved by PVP freezing in liquid nitrogen can maintain their antigenic reactivity to common blood group antigens comparable to that of fresh red cells [12, 19, 20, 21]. We studied 8 patients for a set of 22 antigens (Tables S3 and S4, https://doi.org/10.48623/aperta.286319), and corroborated that our bulk freezing with PVP preserved the antigen expression necessary for serologic testing after thawing, as compared with the molecular grouping results.

Our real-world data from a modern transfusion medicine setting aligns with previous knowledge from the 1970s [2,4–7,10,17–20]. Optimization of incubation and thawing parameters could further enhance red cell recovery but may not be required for practical purposes in the clinical testing laboratory. Laboratories may adopt a method with lower recovery rate because the overall amount of recovered red cells may exceed that of droplet freezing while reducing the required storage capacity. The new addition to the toolkit may be particularly useful for antibody identification requiring adsorption/elution studies. Bulk freezing with PVP in liquid nitrogen offers a practical and effective method for large-volume cryopreservation of red cells.

## Supporting information

Additional supporting information is in the online version of this article:

**Table S1:** Freezing and thawing characteristics and recovery rate of red cell samples after processing of patient and donor samples.

Sample number	Patient or donor	Sex	Time from collection to freezing (days)	Incubation method	Thawing temperature	Recovery rate (%)
1	P	M	24 days	MA	42 °C	43%
2	P	M	21 days	NA	42 °C	37%
3	P	M	24 days	MA	37 °C	55%
4	P	M	21 days	NA	37 °C	36%
5	P	F	24 days	MA	37 °C	48%
6	P	F	21 days	NA	37 °C	47%
7	P	M	24 days	MA	37 °C	46%
8	P	F	21 days	NA	37 °C	63%
9	P	M	27 days	MA	37 °C	49%
10	P	F	21 days	NA	37 °C	40%
11	P	M	24 days	MA	37 °C	53%
12	P	F	21 days	NA	37 °C	48%
13	P	M	24 days	MA	37 °C	41%
14	P	F	21 days	NA	37 °C	39%
15	P	M	23 days	MA	37 °C	53%
16	P	F	21 days	NA	37 °C	43%
17	P	F	27 days	MA	37 °C	44%
18	P	F	21 days	NA	37 °C	37%
19	P	F	24 days	MA	37 °C	36%
20	P	F	21 days	NA	37 °C	31%
21	P	M	25 days	MA	22 °C	36%
22	P	F	21 days	NA	37 °C	43%
23	P	F	27 days	MA	37 °C	48%
24	P	M	21 days	NA	37 °C	50%
25	P	F	25 days	MA	37 °C	56%
26	P	F	21 days	NA	42 °C	43%
27	P	F	24 days	MA	40 °C	41%
28	P	F	21 days	NA	37 °C	50%
29	P	M	24 days	MA	37 °C	46%
30	P	M	21 days	NA	42 °C	40%
31	P	F	25 days	MA	37 °C	81%
32	P	F	21 days	NA	37 °C	37%
33	P	M	24 days	MA	42 °C	44%
34	P	M	21 days	NA	42 °C	35%
35	P	F	27 days	MA	37 °C	47%
36	P	F	21 days	NA	37 °C	60%
37	P	F	24 days	MA	37 °C	45%
38	P	F	21 days	NA	22 °C	34%
39	P	M	25 days	MA	37 °C	55%
40	P	M	21 days	NA	42 °C	35%
41	P	F	25 days	MA	37 °C	45%
42	P	M	21 days	NA	37 °C	45%
43	P	M	25 days	MA	42 °C	49%
44	P	F	21 days	NA	37 °C	54%
45	P	F	21 days	NA	37 °C	48%
46	P	F	21 days	NA	37 °C	44%
47	P	M	24 days	MA	37 °C	54%
48	P	M	21 days	NA	22 °C	32%
49	D	U	2 days	NA	37 °C	35%
50	D	U	3 days	NA	45 °C	42%
51	D	U	2 days	NA	50 °C	34%
52	D	U	2 days	MA	37 °C	34%
53	D	U	3 days	MA	37 °C	41%
54	D	U	2 days	NA	37 °C	37%

D – Donor

MA –Slow agitation

NA – No agitation

P – Patient

U – Undisclosed

**Table S2:** Combined effects of incubation method and thawing temperature on red cell recovery analyzed by a generalized linear model.

Predictors	Estimate (B)	Standard error	95% CI (Lower – Upper)	df	p
Intercept	43.80	3.14	37.65–49.96	1	<0.001
Incubation method					
NA vs. MA	−4.88	2.24	−9.28 – −0.49	1	0.029
MA (reference)	–	–	–		–
Thawing temperature					
22 °C vs. 42 °C	−6.55	5.38	−17.09–3.99	1	0.224
37 °C vs. 42 °C	+5.22	3.09	−0.84–11.27	1	0.091

df – Degrees of freedom

MA – Mild agitation

NA – No agitation

**Table S3:** Antigen detection with serologic methods after red cell thawing: blood group systems ABO, RH, and KEL.

Patient Number			RH						KEL					
	Incubation	ABO *	D	C	c	E	e	V	K	k	Kp ^a^	Kp ^b^	Js ^a^ †	Js ^b^
11	NA	A	Pos	Pos	Neg	Neg	Pos	Neg	Neg	Pos	Neg	Pos	Neg	Pos
12	MA	O	Pos	Pos	Pos	Neg	Pos	Pos	Neg	Pos	Neg	Pos	Neg	Pos
14	NA	O	Pos	Neg	Pos	Pos	Pos	Neg	Neg	Pos	Neg	Pos	Neg	Pos
24	MA	O	Pos	Pos	Pos	Neg	Pos	Neg	Neg	Pos	Neg	Pos	Neg	Pos
28	MA	A	Pos	Pos	Neg	Neg	Pos	Neg	Neg	Pos	Pos	Pos	Neg	Pos
37	NA	O	Pos	Neg	Pos	Pos	Pos	Neg	Pos	Pos	Neg	Pos	Neg	Pos
46	NA	O	Pos	Pos	Pos	Neg	Pos	Neg	Neg	Pos	Neg	Pos	Neg	Pos
50	MA	B	Pos	Neg	Pos	Pos	Pos	Neg	Pos	Pos	Neg	Pos	Neg	Pos
Antigen typing														
Antibody: mono/polyclonal				mono	mono	mono	mono	poly	mono	mono	poly	poly	n.a.	poly
Clone used				MS24	MS42	C2	MS16	-	MS56	P3A118OL67	n.a.	n.a.	n.a.	n.a.
IgM/IgG				IgM	IgM	IgM	IgM	IgG	IgG	IgG	IgG	IgG	n.a.	IgG
Test phase				IS	IS	IS	IS	AHG	AHG	AHG	AHG	AHG	n.a.	AHG
Incubation temperature				RT	RT	RT	RT	37 °C	37 °C	37 °C	37 °C	37 °C	n.a.	37 °C
Incubation time				n.a.	n.a.	n.a.	n.a.	15 min	15 min	15 min	30 min	30 min	n.a.	15 min
Method used				tube	tube	tube	tube	tube	gel	tube	gel	tube	n.a.	tube
Manufacturer				Ortho	Ortho	Ortho	Ortho	n.a.	Ortho	Immucor	Immucor	Immucor	n.a.	ARC

The table shows all molecular grouping results of the patients and yellow boxes show serologic typing of molecularly antigen-positive cells after thawing

* Only serologic results for ABO antigens

† The Js^a^ antigen was not tested, because no patient was predicted to express Js^a^.

AHG – Antihuman globulin

IS – Immediate spin

MA – Mild agitation

NA – No agitation

n.a. – Not applicable

RT – Room temperature

**Table S4:** Antigen detection with serologic methods after red cell thawing: blood group systems, other than ABO, RH, and KEL.

Patient Number		Fy		Jk		MNS					Lu	
	Incubation	Fy ^a^	Fy ^b^	Jk ^a^	Jk ^b^	M	N	S	s	U	Lu ^a^	Lu ^b^
11	NA	Neg	Pos *	Pos	Pos	Neg	Pos	Neg	Pos	Pos	Neg	Pos
12	MA	Neg	Pos	Pos	Pos	Pos	Pos	Neg	Pos	Pos	Neg	Pos
14	NA	Pos	Pos *	Pos	Pos	Pos	Pos	Neg	Pos	Pos	Neg	Pos
24	MA	Pos	Neg	Neg	Pos	Pos	Pos	Pos	Pos	Pos	Pos	Pos
28	MA	Pos	Pos	Neg	Pos	Pos	Neg	Pos	Pos	Pos	Neg	Pos
37	NA	Pos	Pos	Pos	Neg	Pos	Neg	Neg	Pos	Pos	Neg	Pos
46	NA	Pos	Neg	Pos	Neg	Pos	Neg	Pos	Neg	Pos	Neg	Pos
50	MA	Pos	Pos	Pos	Neg	Pos	Pos	Pos	Pos	Pos	Neg	Pos
Antigen typing												
Antibody:mono/polyclonal		mono	poly	mono	mono	mono	mono	mono	mono	poly	poly	mono
Clone used		P3TIM	n.a.	MS-15	MS-8	M2A1	12E.A1	MS-94	P3BER	n.a.	n.a.	LU2
IgM/IgG		IgG	IgG	IgM	IgM	IgG1	IgG1	IgM	IgM	IgG	IgG	IgG
Test phase		AHG	AHG	RT	RT	RT	RT	RT	RT	AHG	AHG	AHG
Incubation temperature		37 °C	37 °C	RT	RT	RT	RT	RT	RT	37 °C	37 °C	37 °C
Incubation time		15 min	15 min	15 min	15 min	15 min	15 min	15 min	15 min	15 min	15 min	15 min
Method used		tube	tube	tube	tube	tube	tube	tube	tube	tube	gel	tube
Manufacturer		Immucor	Ortho	Immucor	Immucor	Immucor	Immucor	Immucor	Immucor	n.a.	n.a.	Albaclone

* Fy^b^ antigen positive, mixed field reactions in transfused patients no 11 and 14

The table shows all molecular grouping results of the patients and yellow boxes show serologic typing of molecularly antigen-positive cells after thawing

AHG – Antihuman globulin

IS – Immediate spin

MA – Mild agitation

NA – No agitation

n.a. – Not applicable

RT – Room temperature

## Figures and Tables

**Table 1:** Red cell recovery after PVP bulk freezing. The results show the effects of different incubation methods and thawing temperatures.

Variables	Red cell samples(n)	Recovery (%) (mean ± SD)	p
Incubation*			
No agitation	27	42.3% ± 8.1%	0.017†
Slow agitation	24	47.9% ± 9.3%	
Total	51		
Thawing temperature			
22 °C	3	34.0% ± 2.0%	
37 °C	40	46.6% ± 9.3%	0.007‡
42 °C	8	40.8% ± 4.9%	
Total§	51		

* 1 h at room temperature

§ 3 samples of 40 °C, 45 °C, and 50 °C were excluded

† Mann–Whitney U test, 2-tailed

‡ Kruskal–Wallis test, 2-tailed

n: number of patients

SD: standard deviation

**Table 2:** Red cell recovery after PVP bulk freezing. Note, 3 samples of 40 °C, 45 °C, and 50 °C were excluded.

Incubation condition	Recovery rate (%) and thawing temperature					
	22 °C		37 °C		42 °C	
	mean ± SD	n	mean ± SD	n	mean ± SD	n
No agitation	33.0% ± 1.4%	2	44.3% ± 8.4%	20	38.0% ± 3.5%	5
Slow agitation	36.0%	1	48.9% ± 9.8%	20	45.3% ± 3.2%	3

n: number of patients

SD: standard deviation

## References

[1] Smith AU Prevention of haemolysis during freezing and thawing of red blood-cells Lancet 1950 2 6644 910 911 10.1016/s0140-6736(50)91861-7 14795743

[2] Scott KL Lecak J Acker JP Biopreservation of red blood cells: past, present, and future Transfus Med Rev 2005 19 2 127 142 10.1016/j.tmrv.2004.11.004 15852241

[3] Meryman HT Cryoprotective agents Cryobiology 1971 8 2 173 183 10.1016/0011-2240(71)90024-1 5578883

[4] Bricka M Bessis M Preservation of erythrocytes by freezing in the presence of polyvinylpyrrolidone and dextran Completes Rendus des Seances de la Societe de Biologie et de ses Filiales 1955 149 9–10 875 877 13270460

[5] Steinbuch M Quentin M Recherches sur la congélation du globule rouge en milieu macromoléculaire Vox Sanguinis 1958 3 2 123 142 10.1111/j.1423-0410.1958.tb03604.x 13544236

[6] Persidsky M Richards V Mode of protection with polyvinylpyrrolidone in freezing of bone marrow Nature 1962 196 4854 585 586 10.1038/196585a0

[7] Richards V Braverman M Floridia R Persidsky M Lowenstein J Initial clinical experiences with liquid nitrogen preserved blood, employing PVP as a protective additive American Journal of Surgery 1964 108 313 322 10.1016/0002-9610(64)90025-X 14195228

[8] Mosbah IB Franco-Gou R Abdennebi HB Hernandez R Escolar G Effects of polyethylene glycol and hydroxyethyl starch in University of Wisconsin preservation solution on human red blood cell aggregation and viscosity Transplantation Proceedings 2006 38 5 1229 1235 10.1016/j.transproceed.2006.02.068 16797270

[9] Pellerin-Mendes C Million L Marchand-Arvier M Labrude P Vigneron C In vitro study of the protective effect of trehalose and dextran during freezing of human red blood cells in liquid nitrogen Cryobiology 1997 35 2 173 186 10.1006/cryo.1997.2038 9299109

[10] Luyet B Rapatz G A review of basic researches on the cryopreservation of red blood cells Cryobiology 1970 6 5 425 482 10.1016/s0011-2240(70)80101-8 4912860

[11] Pegg DE Diaper MP Scholey SE Coombs RR Cryopreservation of antibody-coupled red cells for use in immunoassays Journal of Immunological Methods 1982 54 3 331 341 10.1016/0022-1759(82)90317-9 6184415

[12] Schmid P Huvard MJ Lee-Stroka AH Lee JY Byrne KM Red blood cell preservation by droplet freezing with polyvinylpyrrolidone or sucrose-dextrose and by bulk freezing with glycerol Transfusion 2011 51 12 2703 2708 10.1111/j.1537-2995.2011.03258.x 21790629 PMC3470803

[13] Lovelock JE The protective action of neutral solutes against haemolysis by freezing and thawing Biochemical Journal 1954 56 2 265 270 10.1042/bj0560265 13140185 PMC1269611

[14] Meryman HT Modified model for the mechanism of freezing injury in erythrocytes Nature 1968 218 5139 333 336 10.1038/218333a0 5649669

[15] Takahashi T Hirsh A Erbe E Williams RJ Mechanism of cryoprotection by extracellular polymeric solutes Biophysical Journal 1988 54 3 509 518 10.1016/s0006-3495(88)82983-7 2462928 PMC1330349

[16] Ishiguro H Rubinsky B Mechanical interactions between ice crystals and red blood cells during directional solidification Cryobiology 1994 31 5 483 500 10.1006/cryo.1994.1059 7988158

[17] Doebbler GF Rinfret AP The influence of protective compounds and cooling and warming conditions on hemolysis of erythrocytes by freezing and thawing Biochimica and Biophysica Acta 1962 58 3 449 458 10.1016/0006-3002(62)90055-0 13886879

[18] Sakaida RR Doebbler GF Kibler RS Rinfret AP Rapid freezing and thawing of blood Annals of the New York Academia of Sciences 1965 125 2 647 657 10.1111/j.1749-6632.1965.tb45419.x 5221085

[19] Huntsman RG Hurn BA Lehmann H Storage of red cells for blood-grouping after freezing in liquid nitrogen British Medical Journal 1960 2 5192 118 10.1136/bmj.2.5192.118 14405486 PMC2096786

[20] Bronson WR Mc GM The preservation of human red blood cell agglutinogens in liquid nitrogen: study of a technic suitable for routine blood banking Blood 1962 20 478 484 13873426

[21] Chagas MA Chaves DG Haddad SK Ubiali EM Schmidt LC Effect of red blood cell preservation by droplet freezing with non-permeable cryoprotective agents in blood group antigen reactivity Transfusion Medicine 2017 27 2 142 146 10.1111/tme.12385 28111825

[22] Meryman HT Hornblower M A simplified procedure for deglycerolizing red blood cells frozen in a high glycerol concentration Transfusion 1977 17 5 438 442 10.1046/j.1537-2995.1977.17578014580.x 910260

[23] O’Connor JEL Identification of antibodies to red cell antigens Cohn CSDM Johnson ST Katz LM Schwartz J AABB Technical Manual 21st edition Bethesda MD AABB Press 2023 435 437

